# Molecular epidemiology and pathological burden of *Mycobacterium avium* subsp. *paratuberculosis* in staple livestock from Pakistan: implications for zoonotic surveillance and livestock productivity

**DOI:** 10.1128/spectrum.02890-25

**Published:** 2026-10-02

**Authors:** Farhan Anwar Khan, Salman Khan, Muhammad Saeed, Toufeeq ur Rehman, Arshad Zahoor, Khadija Tul Kubra, Rajwali Khan

**Affiliations:** 1 College of Veterinary Sciences, Faculty of Animal Husbandry and Veterinary Sciences, The University of Agriculture 66936, Peshawar, Pakistan; 2 Department of Livestock Management, Faculty of Animal Husbandry and Veterinary Sciences, The University of Agriculture 66936, Peshawar, Pakistan; Kwame Nkrumah University of Science and Technology College of Health Sciences, Kumasi, Ghana; China Agricultural University, Beijing, China; Xavier University School of Medicine, Aruba, Aruba

**Keywords:** paratuberculosis, MAP, *M. bovis*, One Health, abattoir surveillance

## Abstract

Paratuberculosis (Johne’s disease), caused by *Mycobacterium avium* subsp. *paratuberculosis* (MAP), is a chronic granulomatous enteritis affecting domestic and wild ruminants worldwide, with significant economic losses. Its potential zoonotic implications in humans have increased concern globally. Despite its global importance, limited data exist on MAP prevalence in Pakistan’s Khyber Pakhtunkhwa province. A cross-sectional study was conducted in Peshawar district, Pakistan, using a multimodal diagnostic approach. Serum (*n* = 350), fecal (*n* = 350), and tissue samples (intestine and mesenteric lymph nodes; *n* = 400) were collected from cattle and buffaloes. Samples were analyzed using indirect enzyme-linked immunosorbent assay, Ziehl-Neelsen staining, histopathology, and PCR targeting IS900, IS1311, and *Mycobacterium bovis*-specific genes. Seroprevalence of MAP was 9% (17/175) in cattle and 4% (7/175) in buffaloes (*P* = 0.03). Acid-fast bacilli were detected in 22% (39/175) of cattle and 10% (19/175) of buffalo fecal samples. Gross pathological lesions, including thickness/corrugation in the intestine and hemorrhages/edematous swelling in the mesenteric lymph node, were found at higher frequencies in cattle compared to buffaloes. PCR confirmed MAP in 5% of cattle and 3% of buffalo tissue samples, while one cattle sample harbored *M. bovis*. Emaciated animals exhibited significantly higher lesion severity (*P* < 0.001). This study confirms endemic MAP infection in staple livestock of the study area, with higher susceptibility in cattle. This study underscores the importance of molecular diagnostics in surveillance and the critical role of abattoirs in monitoring disease prevalence. The presence of MAP and co-detection of *M. bovis* highlight the need for integrated control strategies under a One Health framework to investigate and mitigate potential zoonotic risks and production losses in resource-limited dairy systems.

**IMPORTANCE:**

This study provides the first comprehensive molecular epidemiological data on *Mycobacterium avium* subsp. *paratuberculosis* (MAP) in cattle and buffaloes from Khyber Pakhtunkhwa, Pakistan. Using a multimodal approach (serology, histopathology, and PCR), it reveals significant infection rates, with cattle being more susceptible than buffaloes. The work is critically significant as it confirms the endemic presence of MAP in a key dairy region and highlights a concurrent infection with *Mycobacterium bovis*, underscoring a compounded potential zoonotic risk. These findings emphasize the urgent need for integrated One Health strategies to mitigate transmission through improved surveillance, milk pasteurization, and control programs in resource-limited settings.

## INTRODUCTION

Paratuberculosis, or Johne’s disease, is a chronic progressive enteritis caused by *Mycobacterium avium* subsp. *paratuberculosis* (MAP). The disease affects a wide range of ruminants, including cattle, buffaloes, sheep, and goats, leading to substantial economic losses due to progressive weight loss, chronic diarrhea, weight loss, decreased productivity, infertility, and premature culling (1, 2). Globally, MAP infection results in annual economic losses exceeding USD 1.5 billion in the dairy industry alone. The estimated losses are due to a 15%–25% reduction in milk yield (2), 10%–15% premature culling rates and reproductive failures, and diminished carcass value (3). Beyond its veterinary impact, MAP is implicated in human Crohn’s disease, a chronic inflammatory bowel disorder, raising significant public health concerns (4–7). Viable MAP has been isolated from Crohn’s disease patients, and molecular studies confirm the presence of MAP-specific DNA in intestinal tissues of affected individuals (7).

MAP is a hardy, slow-growing mycobacterium with a unique ability to survive in the environment for extended periods (up to 12 months in soil and water) and withstand standard pasteurization temperatures (4, 8). The pathogen enters the host via the fecal-oral route, primarily infecting young animals, though clinical signs typically manifest in adults aged 3–5 years. Following ingestion, MAP is phagocytosed by M cells and macrophages in the ileum, where it disrupts phagolysosome maturation and survives intracellularly, leading to granulomatous inflammation and eventual tissue destruction (9). The prolonged subclinical phase, during which infected animals shed MAP and other pathogens intermittently in feces and milk, complicates diagnosis and control (10, 11).

Diagnosis of MAP infection remains challenging due to its long incubation period, intermittent shedding, and the limitations of individual tests. Serological methods like enzyme-linked immunosorbent assay (ELISA) detect antibodies during advanced infection but lack sensitivity in subclinical stages (12, 13). Direct detection methods include culture (gold standard but time-consuming), acid-fast staining (low sensitivity), and molecular techniques such as PCR, which offer high specificity and rapid results (8, 14). Histopathological evaluation of affected tissues remains a gold standard for confirming granulomatous enteritis but requires post-mortem samples. However, no single test offers optimal sensitivity across all disease stages, making combined diagnostic approaches essential for effective surveillance (15–18). *Mycobacterium bovis*, the causative agent of bovine tuberculosis, is another major zoonotic mycobacterium with significant public health and economic implications in many developing countries (19).

In Pakistan, livestock plays a vital role in the economy (20), contributing approximately 62% to the agricultural GDP and supporting 8 million rural families (Pakistan Economic Survey, 2025). Despite its significance, comprehensive MAP surveillance remains limited and fragmented in Pakistan, particularly in the Khyber Pakhtunkhwa (KPK) province. Previous studies in Punjab province reported prevalence rates of 4.5%–22.6% in cattle and buffaloes (14, 21, 22), but no comprehensive studies exist for KPK, a major dairy-producing region. Peshawar, the capital of KP, hosts dense livestock populations with intensive farming practices, creating optimal conditions for MAP transmission. Furthermore, the consumption of raw milk and traditional dairy products in the region poses a direct zoonotic risk. This study was conducted to evaluate the prevalence of MAP in cattle and buffaloes in District Peshawar through an integrated diagnostic approach. The findings aim to inform evidence-based interventions to control MAP and safeguard both livestock productivity and public health.

## MATERIALS AND METHODS

### Study area and design

**Fig 1:**
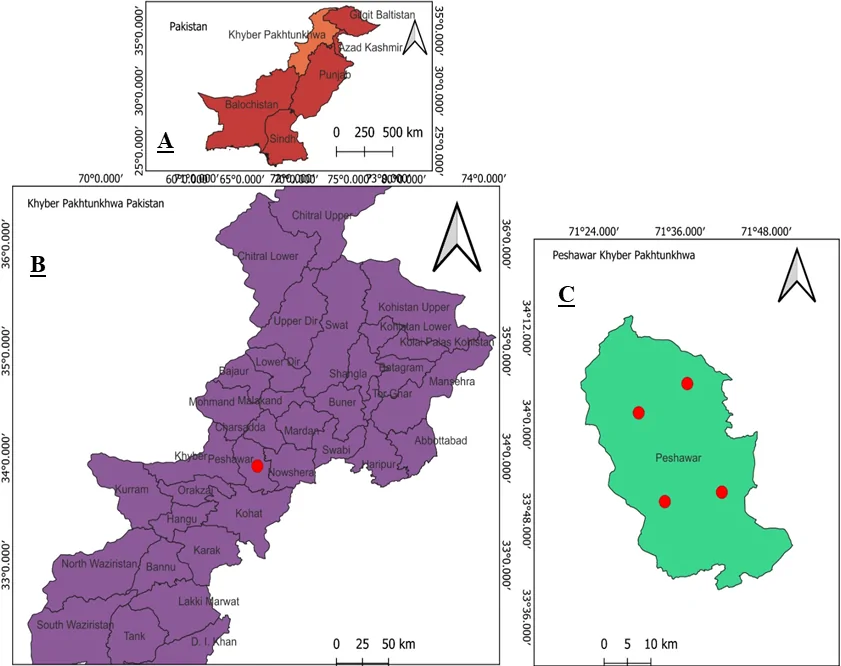
Geographical context of the study area: (**A**) Map of Pakistan. (**B**) Map of Khyber Pakhtunkhwa province, with district Peshawar highlighted (red circle). (**C**) Map of Peshawar district showing sampling locations. The maps were generated using QGIS version 3.44.8-Solothurn.

A cross-sectional study was conducted in Peshawar district (34.015°N, 71.524°E), Khyber Pakhtunkhwa, Pakistan (Fig. 1). The district has a semi-arid climate with an average annual rainfall of 400 mm and temperatures ranging from 2°C (winter) to 45°C (summer). Livestock farming is predominantly smallholder-based, with mixed dairy (cattle and buffalo) production systems.

### Sample collection

A stratified random sampling approach was employed. Samples were collected from commercial dairy farms (*n* = 20), randomly selected from four administrative tehsils (subdivisions) and abattoirs (*n* = 3), representing major slaughter facilities in Peshawar. All sample collections were performed following approved biosafety protocols to minimize infection risk to personnel and environmental contamination.

Sample types and size include blood samples (*n* = 350: 175 cattle and 175 buffaloes) that were collected via jugular venipuncture using sterile, single-use needles and vacutainer tubes, centrifuged at 3,000 × *g* for 10 min. The separated sera were then aliquoted into sterile microcentrifuge tubes and stored at −20°C for a maximum of 3 weeks before being tested (23). Short-term storage at this temperature is within the manufacturer’s recommendations for the IDvet ELISA kit and is therefore unlikely to have affected antibody stability. Fresh fecal samples (*n* = 350: 175 cattle and 175 buffaloes) were collected directly from the rectum using sterile gloves and containers and transported on ice (Table 1). Tissue samples included intestinal segments (ileocecal junction) and mesenteric lymph nodes (MLN; *n* = 400: 200 cattle and 200 buffaloes) were collected using sterile surgical instruments during routine slaughter (Table 1), fixed in 10% neutral buffered formalin for histopathology (24), and stored at −86°C for PCR.

**TABLE 1:** Samples collected from large ruminants at district Peshawar^*a*^

Matrix	Cattle	Buffaloes	Total	Source
Serum	175	175	350	Farms/abattoirs
Feces	175	175	350	Farms/abattoirs
Intestinal	100	100	200	Abattoirs
MLN	100	100	200	Abattoirs

^*a*^ MLN, mesenteric lymph nodes.

Moreover, all biological samples were considered potentially infectious and handled using appropriate personal protective equipment, including gloves, lab coats, and face shields. Samples were transported to the laboratory in leak-proof primary containers with absorbent material, placed inside sealed secondary packaging, and clearly labeled according to institutional biosafety guidelines for infectious materials. Laboratory processing was conducted under biosafety level 2 conditions. The study protocol was reviewed and approved by the Institutional Biosafety Committee.

### Laboratory analyses

#### Indirect ELISA

Serum samples were tested using a commercial indirect ELISA (iELISA) kit (ID Screen Paratuberculosis, IDvet, Grabels, France) following the manufacturer’s protocols. Optical density was read at 450 nm. Samples with sample-to-positive (S/P) ratios ≥ 0.4 were considered positive (13).

#### Ziehl-Neelsen staining

Fecal impression smears were prepared, fixed, and stained with carbol fuchsin, decolorized with acid-alcohol, and counterstained with methylene blue. Acid-fast bacilli (AFB) were identified as bright red rods under oil immersion (1,000× magnification) (14).

#### Histopathology

Formalin-fixed tissues were processed, embedded in paraffin, sectioned at 5 µm, and stained with hematoxylin and eosin (H&E). Lesions were graded using the González staging system: Stage I: focal granulomas in lamina propria; Stage II: transmural lymphoid infiltration; and Stage III: diffuse necrosis with epithelioid macrophages and Langhans giant cells (25, 26).

#### DNA extraction and PCR

Genomic DNA was extracted from tissue samples using the Tissue Kit (Thermo, USA). PCR amplification was performed in a Bio-Rad thermal cycler using primers targeting: MAP IS900 (626 bp), MAP IS1311 (550 bp), and *M. bovis* (500 bp) (Table 2). Cycling conditions: initial denaturation at 94°C for 5 min; 35 cycles of 94°C (1 min), 55°C (30 s), 72°C (1 min); final extension at 72°C for 10 min. Amplicons were visualized on 1.5% agarose gels (27–29).

**TABLE 2:** Sets of primers used for PCR analysis of intestinal tissue infected with MAP and *M. bovis*

Target	Primers (5′−3′)	Amplicon
IS900	F: GTTATTAACGACGCCCAGC	626 bp
	R: ACGATGCTGTGTTGGGCGTTAG	
IS1311	F: TGAACGGAGCGCATCACGAA	550 bp
	R: TGCAGCTGGTGATCTCTGAT	
*M. bovis*	F: TCGTCCGCTGATGCAAGTGC	500 bp
	R: CGTCCGCTGACCTCAAGAAG	

### Statistical analysis

Data were analyzed using SPSS v25.0 (IBM Corp., USA). Seroprevalence and tissue prevalence were compared using Chi-square or Fisher’s exact test. Risk factors associated with MAP infection were assessed using multivariable logistic regression, with iELISA seropositivity (S/P ratio ≥0.4) as the dependent variable. Serology was chosen as the primary outcome for risk factor analysis as it captures infection status across all disease stages, provided the largest sample size (*n* = 350), and aligns with standard epidemiological approaches for MAP risk factor assessment. Ziehl-Neelsen (ZN) staining and PCR results were analyzed descriptively and used for diagnostic confirmation but were not included as primary outcomes in the risk factor model due to smaller sample sizes (*n* = 200 for PCR, *n* = 350 for ZN with lower specificity) and the focal nature of tissue-based detection.

Multivariable logistic regression was performed to assess risk factors associated with MAP infection, with seropositivity (iELISA) as the dependent variable. The initial model included the following independent variables: species (cattle vs buffalo), sample source (farm vs abattoir), body condition score (emaciated vs normal), and management system (zero-grazing vs semi-intensive). Confounding was assessed using a change-in-estimate criterion of ≥10%. Age and sex were evaluated as potential confounders but were not retained in the final model as they did not substantially alter the effect estimates (<10% change) and were not independently associated with the outcome. Histopathological lesion severity was correlated with body condition using Spearman’s rank correlation. Significance was set at *P* < 0.05.

## RESULTS

### Seroprevalence of MAP

Overall MAP seroprevalence was 6.85% (24/350), with significantly higher rates in cattle (9.0%, 17/175) than buffaloes (4.0%, 7/175) (*P* = 0.03) (Table 3). Seropositivity was higher in abattoir-derived samples (8.0%, 14/175) compared to farm samples (5.70%, 10/175) (*P* = 0.008) (Table 4).

**TABLE 3:** Diagnostic confirmation of MAP using a variety of samples and techniques

Method	Cattle	Buffaloes	*P* value
iELISA (serum)	9.0% (17/175)	4.0% (7/175)	0.03
ZN (feces)	22.0% (39/175)	10.0% (19/175)	<0.001
PCR (tissues)	5.0% (5/100)	3.0% (3/100)	0.31

**TABLE 4:** Serological diagnosis of MAP using samples from various locations

Location	iELISA (serum sample)			*P* value
	Cattle	Buffaloes	Percentage	
Farm	7	3	5.70% (10/175)	0.008
Abattoir	10	4	8.0% (14/175)	

### Detection of acid-fast bacilli

**Fig 2:**
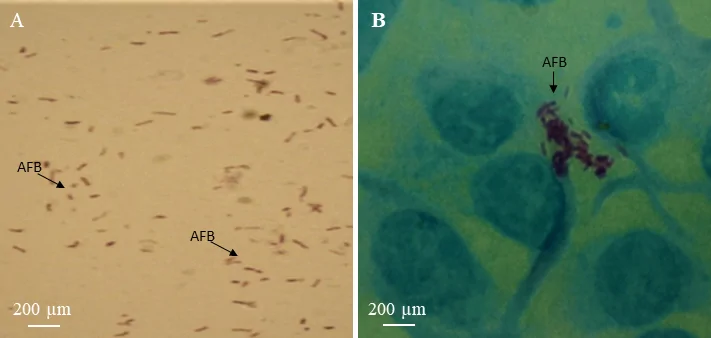
Representative photomicrographs of AFB: (**A**) an intestinal tissue sample from a PCR-positive cow and (**B**) a fecal impression smear from a different cow with positive ZN staining; scale bar = 200 µm.

ZN staining revealed AFB in 16.57% (58/350) of fecal samples, with higher rates in cattle (22.0%, 39/175) than buffaloes (10.0%, 19/175) (*P* < 0.001) (Table 3). AFB of red-rose color were examined in tissue and fecal samples under the microscope (Fig. 2).

### Pathological lesions in tissue samples

**Fig 3:**
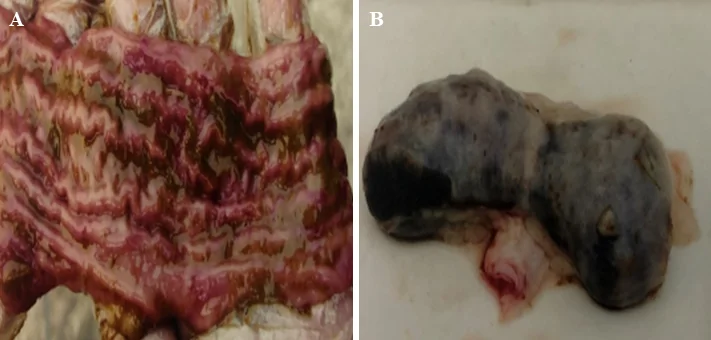
Gross-pathological lesions observed in tissue samples from cow: (**A**) hemorrhage and corrugation in the intestinal mucosa and (**B**) edematous swelling and congestion in MLN.

Tissue samples including intestinal (ileo-caecal junction) and MLN from both cattle and buffaloes (Table 1) were examined for gross lesions and histopathological lesions. Out of 100 cattle, 45% of animals were emaciated, scored on the basis of atrophy of body fat, and 55% were in normal body condition. Diarrhea was noticed in 19.5% of emaciated animals. After slaughtering, intestines from animals (35%) and MLN from 40% of animals exhibited various gross pathologies, i.e., hemorrhages, mucosal thickening or corrugation (Fig. 3A), congestion, and edematous swelling (ES; Fig. 3B) (Table 5). In a total of 100 cattle, emaciation was observed in 45% of cattle. Only six emaciated cattle were suffering from diarrhea. Intestinal samples from 25% and MLN from 32% of animals showed various gross lesions (Table 5).

**TABLE 5:** Apparent body condition and gross lesion of various degrees in tissue samples of examined animals^*a*^

Animal species	Body condition/no. of animals (%)	Tissue	Tissue with gross lesion (%)	Gross lesions	Tissue samples having gross lesions of various degrees (%)		
					Mild	Moderate	Severe
Cattle (100)	Emaciated/45 (45)	Intestine MLN	35 (35) 40 (40)	Hg & Cr ES & Cg	7 (20) 9 (22.5)	11 (31.42) 13 (32.5)	17 (48.57) 18 (45)
	Normal/55 (55)	Intestine MLN	0 (0) 0 (0)	Hg & Cr ES & Cg	0 (0) 0 (0)	0 (0) 0 (0)	0 (0) 0 (0)
Buffaloes (100)	Emaciated/35 (35)	Intestine MLN	25 (25) 32 (32)	Hg & Cr ES & Cg	6 (24) 9 (28.12)	6 (24) 12 (37.5)	13 (52) 11 (34.37)
	Normal/65 (65)	Intestine MLN	0 (0) 0 (0)	Hg & Cr ES & Cg	0 (0) 0 (0)	0 (0) 0 (0)	0 (0) 0 (0)

^*a*^ Hemorrhage (Hg) & corrugation (Cr); edematous swelling (ES) and congestion (Cg).

**Fig 4:**
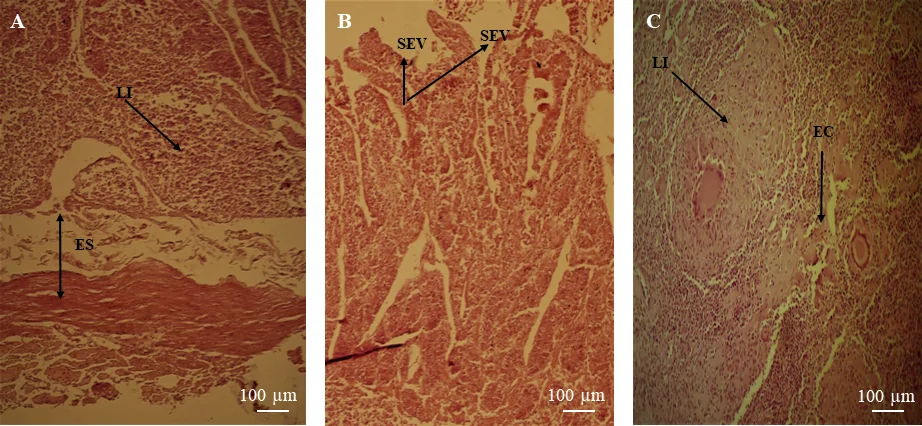
Representative photomicrographs of histopathological lesions recorded in intestinal tissue samples from cattle infected with MAP: (**A**) leukocytic infiltration (LI) in the intestinal villi, ES in the submucosa of the intestine. (**B**) Sloughing of epithelium from intestinal villi (SEV). (**C**) LI and epithelioid cells (EC) in the infected MLN. H&E stain; scale bar = 100 µm.

Histopathologically, granulomatous enteritis characterized by infiltration of epithelioid macrophages, plasma cells, and eosinophils was observed in 28% of cattle intestinal tissues and 22% of MLNs. Buffaloes exhibited similar lesions in 20% and 17% of intestinal and MLN tissues, respectively (Table 6, Fig. 4). Lesion severity was significantly higher in emaciated animals (*P* < 0.001) and correlated with species (cattle > buffaloes, *P* = 0.01).

**TABLE 6:** Histopathological grading (González system)

Species	Stage I	Stage II	Stage III	Total lesions
Cattle	9.0%	12.0%	7.0%	28.0%
Buffaloes	15.0%	5.0%	0.0%	20.0%

### Molecular detection of MAP and *M. bovis*

**Fig 5:**
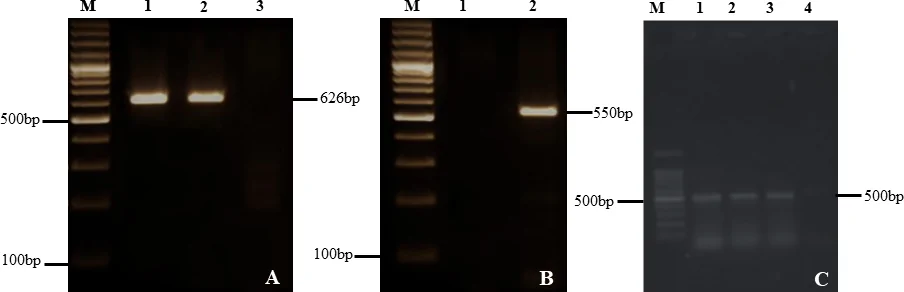
PCR confirmation of MAP and *M. bovis* in tissue samples from different cattle: (M) DNA marker, (**A**1 and 2) an amplicon of 626 bp representing MAP-IS900 detected in cow, (**A**3) negative control, (**B**1) negative control, (**B**2) an amplicon of 550 bp representing MAP-IS1311 detected in the same sample from the same cow for confirmation of MAP, (**C**1–3) an amplicon of 500 bp representing *M. bovis* (triplicate) detection in intestinal tissue sample from different cow, and (**C**4) negative control.

PCR amplification targeting IS900 and IS1311 sequences confirmed MAP DNA in 5 of 100 (4%) cattle and 3 of 100 (3%) buffalo tissue samples. Additionally, one cattle sample tested positive for *M. bovis* using a separate primer set. The *M. bovis*-positive sample was collected from a 6-year-old, emaciated female cow from an abattoir. The animal had gross lesions including caseous necrosis in the mesenteric lymph nodes. MAP was not detected in this animal by PCR or histopathology. No statistically significant difference was observed in molecular detection rates between species (*P* = 0.311). Amplicons of expected sizes (626 bp, 550 bp, and 500 bp) were visualized on agarose gels (Fig. 5, Table 3). We have also performed a Cohen’s kappa analysis to measure agreement between the tests. The *K* = 0.32 for iELISA and PCR, indicating fair agreement.

### Risk factor analysis

Multivariable logistic regression identified several significant risk factors for MAP seropositivity (Table 7). Cattle had 2.38-fold higher odds of MAP infection than buffaloes (OR = 2.38, 95% CI: 1.12–5.05, *P* = 0.02). Abattoir-sourced animals showed 1.72-fold higher odds of seropositivity compared to farm-sourced animals (OR = 1.72, 95% CI: 1.15–2.58, *P* = 0.008). Zero-grazing systems were associated with a 2.12-fold increased infection risk compared to semi-intensive systems (OR = 2.12, 95% CI: 1.18–3.81, *P* = 0.01).

**TABLE 7:** Multivariable logistic regression analysis of risk factors associated with MAP seropositivity (iELISA) in cattle and buffaloes^*a*^

Risk factor	Category	OR	95% CI	*P* value
Species	Cattle vs buffaloes	2.38	1.12–5.05	0.02
Sample source	Abattoir vs farm	1.72	1.15–2.58	0.008
Body condition	Emaciated vs normal	3.20	1.89–5.42	<0.001
Management system	Zero-grazing vs semi-intensive	2.12	1.18–3.81	0.01

^*a*^ OR, odds ratio; CI, confidence interval.

Importantly, emaciated animals exhibited significantly higher lesion severity (3.2-fold, Spearman’s ρ = 0.48, *P* < 0.001). Age and sex were not significantly associated with seropositivity (age: *P* = 0.34, sex: *P* = 0.57) and were not retained in the final model (Table 7). The multivariable model demonstrated adequate fit (Hosmer-Lemeshow *χ*² =6.23, df = 8, *P* = 0.62) (Table 7).

## DISCUSSION

### Epidemiology and diagnostic correlates

This is the first comprehensive study of MAP infection in bovines from KP province, filling a critical geographic data gap in Pakistan. The multimodal approach (serology, ZN staining, histopathology, and multiple PCR targets) on the same population provides a more holistic picture of disease burden than any single-modality study. The findings confirm endemic MAP infection with notable interspecies differences; cattle showed significantly higher seroprevalence (9.0% vs 4.0%), AFB shedding (22.0% vs 10.0%), and histopathological lesions (28.0% vs 20.0%) compared to buffaloes. These results align with global trends where cattle are recognized as more susceptible to MAP than buffaloes (29). The higher susceptibility in cattle may be attributed to anatomical and immunological factors, such as increased ileal M-cell density facilitating bacterial invasion (30) and differential macrophage apoptosis (10). Additionally, management practices in Peshawar (e.g., intensive zero-grazing systems) likely amplify transmission among cattle, as reflected in the 2.12-fold higher infection risk in confined animals (30). The monsoon climate (June–September) prolongs MAP survival in soil (2). Buffaloes’ semi-aquatic behavior (31) may reduce environmental MAP exposure, though recent evidence suggests innate resistance through enhanced macrophage apoptosis (30). The high sensitivity but low specificity of ZN staining explains that it detects AFB, not just MAP. There are several other AFB in the ecological niche.

The diagnostic strategy employed highlighted the strengths and limitations of each method. iELISA detected subclinical infections (seropositivity in 6.85% of animals), though its sensitivity is known to be low in early infection stages (13). PCR provided definitive confirmation of MAP in tissues but showed lower prevalence (4.0%) due to the focal nature of lesions and intermittent shedding (10, 11). Histopathology remains the gold standard for diagnosis, revealing characteristic granulomatous lesions in 24.0% of infected animals. The significant correlation between emaciation and lesion severity (*P* < 0.001) underscores the progressive nature of MAP infection and its economic impact through weight loss and reduced productivity. The different stages of infection detected by each method (e.g., serology detects late-stage humoral response, PCR detects current infection in tissues), and the variability is probably due to sample type (feces vs tissue).

Notably, abattoir-sourced animals showed 1.7-fold higher seropositivity (8% vs farm 4.67%, *P* = 0.008), mirroring global pattern (5, 10), and Pakistani Punjab studies where emaciated culls exhibited three times higher MAP burden (22). Our findings contrast with Punjab’s higher prevalence (11.2%–22.6%) (32), possibly due to Peshawar’s lower stocking density (1.7 animals/household vs Punjab’s 3.2; Livestock Census 2021).

### Comparative global burden

Globally, our reported seroprevalence (6.85%) is lower than hyperendemic regions like Jordan (60%) (33, 34) and Indian Punjab (28.6%) (35) but higher than Chinese Xinjiang (3.7%) (36). This positions Pakistan as moderately endemic, requiring targeted control strategies. The higher prevalence in abattoir-derived samples (8.0% vs farm 4.67%; *P* = 0.008) suggests that clinically advanced animals are culled for slaughter, making abattoirs strategic surveillance points. Climatic factors (MAP survival: humidity > temperature) and husbandry practices explain regional variations (2, 37).

### Zoonotic implications and co-infections

The detection of *M. bovis* in one cattle sample (1.0%) is a critical finding, revealing co-circulation of zoonotic mycobacteria in the region, a compounding risk in regions with raw milk consumption (37). This aligns with recent reports of tuberculosis-paratuberculosis co-infections in resource-limited settings (37). MAP’s confirmed thermal tolerance (9) raises concerns for dairy supply chains, particularly given that 40% of Peshawar’s population consumes raw milk (KP Livestock Department, 2021). The co-detection of *M. bovis* in the same study population, while not co-infection, is a novel finding for the region and highlights the complex zoonotic pathogen landscape. The presence of MAP and *M. bovis* in dairy herds poses direct public health risks. MAP strains isolated from dairy herds in Pakistan show genomic homology with human Crohn’s disease isolates (7), emphasizing the zoonotic potential.

### Limitations and future directions

Our study has several limitations. The cross-sectional, abattoir-based design may introduce selection bias, as sampled animals are more likely to represent a culled or clinically affected population, potentially overestimating true prevalence. The partial longitudinal data preclude analysis of infection dynamics. Furthermore, the absence of detailed herd-level management data (e.g., age, parity, biosecurity practices) limits our ability to identify specific on-farm risk factors. Moreover, culture and sequencing of PCR amplicons were not performed due to resource constraints. Future studies should incorporate milk sampling and molecular typing of MAP strains. It is recommended to implement routine serosurveillance in dairy herds. Promote pasteurization of milk (75°C for 25 s to inactivate MAP). Develop MAP control programs under a One Health framework.

### Conclusion

This study confirms the endemicity of MAP in bovines from Peshawar, Pakistan, with a higher infection burden in cattle than buffaloes. The multimodal approach provided a robust epidemiological assessment for the region. The detection of *M. bovis* in the same livestock population underscores the need for enhanced surveillance of zoonotic pathogens. These findings highlight the critical importance of abattoir-based monitoring and the implementation of evidence-based control strategies to improve livestock productivity. Future research should integrate human and environmental sampling to fully elucidate the One Health dimensions of mycobacterial infections in this setting.

## Supplementary Material

Reviewer comments

## Data Availability

Data supporting this study are available from the corresponding author upon request.

## SUPPLEMENTAL MATERIAL

OPEN PEER REVIEW reviewer-comments.pdf An accounting of the reviewer comments and feedback.

ASM does not own the copyrights to Supplemental Material that may be linked to, or accessed through, an article. The authors have granted ASM a non-exclusive, world-wide license to publish the Supplemental Material files. Please contact the corresponding author directly for reuse.
