# Supplementary material for: Molecular epidemiology and pathological burden of Mycobacterium avium subsp. paratuberculosis in staple livestock from Pakistan: implications for zoonotic surveillance and livestock productivity
Source: Microbiol Spectr. 2026 Oct 2;14(11):e02890-25. doi: 10.1128/spectrum.02890-25 (PMC13729210; doi:10.1128/spectrum.02890-25)
Supplement: Supplementary file 1 [file reviewer-comments.pdf]

ASM | Microbiology Spectrum

Molecular Epidemiology and Pathological Burden of Mycobacterium avium subsp. paratuberculosis in Staple livestock from Pakistan: Implications for Zoonotic Surveillance and Livestock Productivity

Farhan Anwar Khan, Salman Khan, Muhammad Saeed, Toufeeq Rehman, Arshad Zahoor, Khadija Kubra, and Rajwali Khan

Corresponding Author(s): Farhan Anwar Khan, The University of Agriculture Peshawar

Review Timeline:

Submission Date:	September 12, 2025
Editorial Decision:	February 26, 2026
Revision Received:	March 25, 2026
Editorial Decision:	May 8, 2026
Revision Received:	May 12, 2026
Editorial Decision:	June 27, 2026
Revision Received:	June 30, 2026
Accepted:	August 17, 2026

Editor: Michael Owusu

Reviewer(s): Disclosure of reviewer identity is with reference to reviewer comments included in decision letter(s). The following individuals involved in review of your submission have agreed to reveal their identity: Hao Li (Reviewer #1); Ravindra P Veeranna (Reviewer #2)

Transaction Report:

(Note: With the exception of the correction of typographical or spelling errors that could be a source of ambiguity, letters and reports are not edited. The original formatting of letters and referee reports may not be reflected in this compilation.)

DOI: https://doi.org/10.1128/spectrum.02890-25

Re: Spectrum02890-25 (Molecular Epidemiology and Pathological Burden of Mycobacterium avium subsp. paratuberculosis in Staple livestock from Pakistan: Implications for Zoonotic Surveillance and Livestock Productivity)

Dear Dr. Farhan Anwar Khan:

Thank you for the privilege of reviewing your work. Below you will find my comments, instructions from the Spectrum editorial office, and the reviewer comments.

Please return the manuscript within 60 days; if you cannot complete the modification within this time period, please contact me. If you do not wish to modify the manuscript and prefer to submit it to another journal, notify me immediately so that the manuscript may be formally withdrawn from consideration by Spectrum.

Revision Guidelines
To submit your modified manuscript, log into the submission site at https://spectrum.msubmit.net/cgi-bin/main.plex. Go to Author Tasks and click the appropriate manuscript title to begin. The information you entered when you first submitted the paper will be displayed; update this as necessary. Note the following requirements:

• Upload point-by-point responses to the issues raised by the reviewers in a file named "Response to Reviewers," NOT in your cover letter.
• Upload a compare copy of the manuscript (without figures) as a "Marked-Up Manuscript" file.
• Upload a clean .DOC/.DOCX version of the revised manuscript and remove the previous version.
• Each figure must be uploaded as a separate, editable, high-resolution file (TIFF or EPS preferred), and any multipanel figures must be assembled into one file.
• Any supplemental material intended for posting by ASM should be uploaded with their legends separate from the main manuscript. You can combine all supplemental material into one file (preferred) or split it into a maximum of 10 files with all associated legends included.

For complete guidelines on revision requirements, see our Submission and Review Process webpage. Submission of a paper that does not conform to guidelines may delay acceptance of your manuscript.

Data availability: ASM policy requires that data be available to the public upon online posting of the article, so please verify all links to sequence records, if present, and make sure that each number retrieves the full record of the data. If a new accession number is not linked or a link is broken, provide Spectrum production staff with the correct URL for the record. If the accession numbers for new data are not publicly accessible before the expected online posting of the article, publication may be delayed; please contact production staff (Spectrum@asmusa.org) immediately with the expected release date.

Publication Fees: For information on publication fees and which article types are subject to charges, visit our website. If your manuscript is accepted for publication and any fees apply, you will be contacted separately about payment during the production process; please follow the instructions in that e-mail. Arrangements for payment must be made before your article is published.

ASM Membership: Corresponding authors may join or renew ASM membership to obtain discounts on publication fees. Need to upgrade your membership level? Please contact Customer Service at Service@asmusa.org.

The ASM Journals program strives for constant improvement in our submission and publication process. Please tell us how we can improve your experience by taking this quick Author Survey.

Thank you for submitting your paper to Spectrum.

Sincerely,
Michael Owusu
Editor
Microbiology Spectrum

Reviewer #1 (Comments for the Author):

In this study, the authors conducted a cross-sectional study to investigate the MAP prevalence in KPK province in Pakistan, which is a good reference for MAP and M.bovis control in Pakistan.
CommentsL
1: The species name, such as M.bovis, needs to be in italic.

2: Lines 38~40: The authors need to point out the frequencies/
3: Lines 150~151: "Seropositivity was higher in abattoir-derived 150 samples (8.0%, 14/175) compared to farm samples (4.67%, 10/175)". There is no this dataset in table 3.
4: The figure legends need to point out that the results of the figures are representative of the animal samples.
5: The scale bar needs to be added in Figure 4.
6: The authors can draw a map to indicate the location of the sample collection in Pakistan.
7: How about the positive serum or sample prevalence in the dairy farms and slaughter facilities?
8: The authors may show all the positive results of the samples in Figure 3.

Reviewer #2 (Comments for the Author):

The study addresses an important veterinary and public health problem by documenting the prevalence of MAP and co-detection of M. bovis in cattle and buffaloes using a multimodal diagnostic approach (iELISA, ZN staining, histopathology, and PCR). The regional focus on Khyber Pakhtunkhwa (KPK), Pakistan, fills a data gap and supports a One Health perspective. However, the work is largely descriptive, and several claims particularly those related to zoonotic risk and diagnostic performance would benefit from clearer validation and cautious interpretation.
Major Comments
1. Given the discrepancies between:
o iELISA seroprevalence (9% cattle, 4% buffalo),
o ZN positivity (22% cattle 10% Buffalo feces),
o PCR confirmation (5% cattle, 3% buffalo tissue),
the authors should address:
o Possible false positives (e.g., ZN staining is not MAP-specific),
o Stage of infection (subclinical vs clinical),
o Sample type dependent detection variability.
Inclusion of kappa statistics or concordance analysis between tests would strengthen diagnostic interpretation.

2. Serum samples were stored at -20 {degree sign}C, but the duration of storage is not specified. Please justify this storage condition, as -80 {degree sign}C is generally recommended for long-term preservation of antibodies. Prolonged storage at -20 {degree sign}C may affect assay reliability.

3. The manuscript should include the Animal Ethics Committee approval number, not only a general statement of institutional approval.

4. A figure showing PCR amplification data for M. bovis should be included, similar to that provided for MAP.

5. PCR targeting IS900 and IS1311 is appropriate; however: The manuscript should clarify whether sequencing confirmation of PCR amplicons was performed to rule out IS900-like sequences.
o Details on positive and negative controls, contamination prevention, and assay validation are essential.

6. The manuscript reports detection of M. bovis in one sample; however, additional details are required, including: Animal species (cattle or buffalo), Age, sex, and clinical status, Tissue(s) tested and gross lesions (if any),
Whether MAP was simultaneously detected in the same sample.

7. It is unclear whether the PCR, histopathology, and ZN staining images are from the same animal or from different animals. If from different animals, please include representative figures from at least three independent samples for each diagnostic modality to demonstrate reproducibility.

8. As a cross-sectional abattoir-based study, the findings reflect slaughterhouse populations rather than herd-level prevalence. The authors should clearly acknowledge:
o Selection bias,
o Lack of longitudinal data,
o Absence of herd management, age, or production system variables.

9. The regional prevalence data from KPK are valuable; however, the novelty is incremental, as similar MAP prevalence studies using ELISA, PCR, and histopathology have been widely reported globally. The authors should clearly articulate:
o What differentiates this study from prior MAP surveillance studies in South Asia.
o Whether the novelty lies in co-detection of MAP and M. bovis, abattoir-based surveillance, or combined diagnostic modalities.

11. The Introduction should include background information on M. bovis detection in livestock and its zoonotic significance to better contextualize the findings.

12. The study employs multiple diagnostic methods; however, the performance characteristics (sensitivity, specificity, agreement) of each method are not discussed.

13. The abstract emphasizes zoonotic implications and links to Crohn's disease; however:
o No human samples or exposure data were included.
o Claims regarding zoonotic risk should be framed as potential or suggestive, not confirmatory.
o The One Health implications are relevant but should be discussed cautiously.

14. All micrographs should include magnification or scale bars directly on the figure.
Figure legends should clearly state:
o Animal species,
o Tissue type,
o Diagnostic method used,
o Whether the sample was MAP-positive, M. bovis-positive, or co-infected.

15. While the One Health implications are relevant, the conclusions regarding zoonotic risk and links to human disease should be moderated, as no human or environmental samples were analyzed.

Minor Comments
1. Overall language is clear, but some sentences are dense and could be simplified for readability.
2. Ensure consistent formatting of species names (Mycobacterium avium subsp. paratuberculosis, M. bovis).
3. Use consistent terms for sample types (e.g., "tissue samples" vs "intestinal and MLN samples").
4. Clarify whether "seroprevalence" refers strictly to iELISA results.
5. Consider adding "One Health" and "abattoir surveillance" as keywords.
6. The conclusion currently overstates the implications. It should emphasize:
o Endemic presence in livestock,
o Need for improved surveillance,
o Future integration with human and environmental data.

Responses to Comments

We are grateful to the reviewers and the editor for their insightful and constructive comments, which have significantly strengthened our manuscript. We have carefully addressed each point raised. Below is our point-by-point response. All changes in the manuscript have been highlighted using the "Track Changes" feature.

S.No	Reviewer 01 Comments	Responses
1.	The species name, such as M.bovis, needs to be in italic.	Thank you for this observation. We have meticulously reviewed the entire manuscript and ensured that all bacterial species names (e.g., *M. bovis*, *Mycobacterium avium* subsp. *paratuberculosis*) are correctly italicized according to standard taxonomic conventions.
2.	Lines 38~40: The authors need to point out the frequencies/	We agree that the abstract should be more specific. The original sentence has been revised to include the precise frequencies. It now reads: "Seroprevalence of MAP was 9% (17/175) in cattle and 4% (7/175) in buffaloes (p=0.03). Acid-fast bacilli were detected in 22% (39/175) of cattle and 10% (19/175) of buffalo fecal samples."
3.	Lines 152~153: "Seropositivity was higher in abattoir-derived samples (8.0%, 14/175) compared to farm samples (5.70%, 10/175)". There is no this dataset in table 3.	This is a valid point, and we have rectified the omission. We have added a new table i.e Table 4 (page 16) to present the seroprevalence data stratified by source (Farm vs. Abattoir). This makes the data transparent and directly supports the statement in the text.
4.	The figure legends need to point out that the results of the figures are representative of the animal samples.	We have revised all figure legends accordingly. For example, the legend for Figure 1 now states: "Fig. 1: Representative photomicrographs of Acid-fast bacilli (AFB) in (A) an intestinal tissue sample from a PCR-positive cow, and (B) a fecal impression smear from a different cow with positive ZN staining." Similar clarifying statements have been added to the legends of Figures 2, 3, and 4.
5.	The scale bar needs to be added in Figure 4.	A scale bar has been added to Figure 4. We have also updated the

		figure legend to indicate the magnification (e.g., "H&E stain; scale bar = 200 μm"). We also put scale bar on the images. Please refer to Fig.4 (page 22)
6.	The authors can draw a map to indicate the location of the sample collection in Pakistan.	This is an excellent suggestion to enhance the geographical context of the study. We have created a new figure (now Figure 1) showing a map of Pakistan with the Khyber Pakhtunkhwa province and the Peshawar district highlighted. All subsequent figures have been re-numbered accordingly. The figure legend clearly describes the study area.
7.	How about the positive serum or sample prevalence in the dairy farms and slaughter facilities?	As noted in response to comment #3, we have now included this data. The prevalence stratified by source (Farm vs. Abattoir) is now explicitly provided in the Table 4 (page 16) and is also described in the Results section (Lines 152-153).
8.	The authors may show all the positive results of the samples in Figure 3.	While we appreciate the suggestion, space constraints in the manuscript limit the number of individual samples we can show in a single gel image. To address this, we have selected a representative gel image that shows multiple positive samples (A1, A2 for IS900; B2 for IS1311) alongside negative controls to demonstrate the specificity of the assay. The legend has been updated to clarify that these are representative results. All PCR-positive samples were confirmed in at least two independent runs.
	Reviewer 02 Comments	Responses
1.	Given the discrepancies between:<br>• iELISA seroprevalence (9% cattle, 4% buffalo),<br>• ZN positivity (22% cattle 10% Buffalo feces),<br>• PCR confirmation (5% cattle, 3% buffalo tissue),<br>The authors should address:<br>• Possible false positives (e.g., ZN staining is not MAP-specific),	We thank the reviewer for this insightful comment. We have significantly expanded the Discussion (Section 4.1) to address these points directly. We now explicitly discuss:<br>• the high sensitivity but low specificity of ZN staining, explaining that it detects

	• Stage of infection (subclinical vs clinical),<br>• Sample type dependent detection variability.<br>Inclusion of kappa statistics or concordance analysis between tests would strengthen diagnostic interpretation	acid-fast bacilli, not just MAP (line 205-206)<br>• the different stages of infection detected by each method (e.g., serology detects late-stage humoral response, PCR detects current infection in tissues); and 3) the variability due to sample type (feces vs. tissue) (lines 214-216).<br>• We have also performed a Cohen's kappa analysis to measure agreement between the tests. The results (κ = 0.32 for iELISA and PCR, indicating fair agreement) are now included in the Results section (Lines 181-184) and discussed as further evidence of the tests targeting different aspects of infection.
2.	Serum samples were stored at -20 {degree sign}C, but the duration of storage is not specified. Please justify this storage condition, as -80 {degree sign}C is generally recommended for long term preservation of antibodies. Prolonged storage at -20 {degree sign}C may affect assay reliability.	We agree that this is an important methodological detail. The sera were stored at -20°C for a maximum of three weeks before being tested. This short-term storage at -20°C is within the manufacturer's recommendations for the IDvet ELISA kit and is unlikely to have affected antibody stability. We have added this clarification (storage duration) to Section 2.3.1 (Lines 113-116) to justify the condition.
3.	The manuscript should include the Animal Ethics Committee approval number, not only a general statement of institutional approval.	We have added the specific approval number. The statement in Section 10 now reads: "This study was approved by the Advanced Studies and Research Board (ASRB) and the Ethics Committee of The University of Agriculture, Peshawar (Approval No. 73/Director-A&R/2023). All procedures were performed in accordance with the relevant institutional guidelines and regulations." (Line 272-275)

4.	A figure showing PCR amplification data for M. bovis should be included, similar to that provided for MAP	We agree that providing visual data for the *M. bovis* positive sample strengthens the manuscript. A new panel (C) has been added to the PCR figure (now Figure 4) showing the 500 bp amplicon from the *M. bovis*-positive sample (triplicate) alongside a negative control. The figure legend has been updated accordingly.
5.	PCR targeting IS900 and IS1311 is appropriate; however: The manuscript should clarify whether sequencing confirmation of PCR amplicons was performed to rule out IS900-like sequences.<br>Details on positive and negative controls, contamination prevention, and assay validation are essential	These are critical points for ensuring the rigor of molecular diagnostics.<br>We believe that the sets of primers (Table 2) used previously (Stabel et al., 2020; Bannantine et al., 2021; Waters et al., 2021) are highly specific to MAP and M.bovis. As the BLAST analysis at NCBI confirmed 100% identity with reference sequences in GenBank of MAP and M. bovis. We did not go for sequencing due to financial constraints and we use nuclease free water as a negative control in every PCR run to monitor for contamination.
6.	The manuscript reports detection of M. bovis in one sample; however, additional details are required, including: Animal species (cattle or buffalo), Age, sex, and clinical status, Tissue(s) tested and gross lesions (if any), Whether MAP was simultaneously detected in the same sample.	We apologize for the omission and have provided all requested details. The *M. bovis* positive sample was collected from a 6-year-old, emaciated, female cattle from an abattoir. The animal had gross lesions including caseous necrosis in the mesenteric lymph nodes. MAP was not detected in this animal by PCR or histopathology. This information has been added to the Results section (Lines 176-179).
7.	It is unclear whether the PCR, histopathology, and ZN staining images are from the same animal or from different animals. If from different animals, please include representative figures from at least three independent samples for each diagnostic modality to demonstrate reproducibility.	To clarify, the images are from different animals, as is typical for a prevalence study. The figure legends have been revised to state that the images are representative findings from different positive animals.
8.	As a cross-sectional abattoir-based study, the findings reflect slaughterhouse populations rather than herd-	This is an important limitation, and we have now addressed it

	level prevalence. The authors should clearly acknowledge:<br>• Selection bias,<br>• Lack of longitudinal data,<br>• Absence of herd management, age, or production system variables.	explicitly. In the new "Limitations and Future Directions" section (4.4), we state: "Our study has several limitations. The cross-sectional, abattoir-based design may introduce selection bias, as sampled animals are more likely to represent a culled or clinically affected population, potentially overestimating true prevalence. The partial longitudinal data precludes analysis of infection dynamics. Furthermore, the absence of detailed herd-level management data (e.g., age, parity, biosecurity practices) limits our ability to identify specific on-farm risk factors." (lines 242-247)
9.	The regional prevalence data from KPK are valuable; however, the novelty is incremental, as similar MAP prevalence studies using ELISA, PCR, and histopathology have been widely reported globally. The authors should clearly articulate:<br>• What differentiates this study from prior MAP surveillance studies in South Asia.<br>• Whether the novelty lies in co-detection of MAP and M. bovis, abattoir-based surveillance, or combined diagnostic modalities	We thank the reviewer for pushing us to better highlight our study's contribution. We have revised the Introduction and the Discussion (Section 4.1) to more clearly articulate the novel aspects:<br>• This is the first comprehensive study of MAP infection in bovines from KP province, filling a critical geographic data gap in Pakistan.<br>The multimodal approach (serology, ZN staining, histopathology, and multiple PCR targets) on the same population provides a more holistic picture of disease burden than any single-modality study.(lines 191-194),<br>• The co-detection of *M. bovis* in the same study population, while not co-infection, is a novel finding for the region and highlights the complex zoonotic pathogen landscape (236-238).
10.	The Introduction should include background information on M. bovis detection in livestock and its	We have added a sentence to the Introduction (Lines 86-88) to contextualize *M. bovis*. It now

	zoonotic significance to better contextualize the findings	reads: *M. bovis*, the causative agent of bovine tuberculosis, is another major zoonotic mycobacterium with significant public health and economic implications in many developing countries, including Pakistan (19).
11.	The study employs multiple diagnostic methods; however, the performance characteristics (sensitivity, specificity, agreement) of each method are not discussed.	This has been addressed in conjunction with Comment #1. We now discuss the inherent limitations and strengths of each method in the Discussion (Section 4.1) and provide the kappa statistics for inter-test agreement in the Results (Lines 182-184) to give a quantitative measure of their concordance.
12.	The abstract emphasizes zoonotic implications and links to Crohn's disease; however:<br>• No human samples or exposure data were included. o Claims regarding zoonotic risk should be framed as potential or suggestive, not confirmatory.<br>• The One Health implications are relevant but should be discussed cautiously.	This is a crucial point, and we have toned down the language accordingly. The abstract has been thoroughly revised. The phrase "zoonotic link" has been replaced with "potential zoonotic implications (line 30)." The final sentence now reads: "...highlight the need for integrated control strategies under a One Health framework to investigate and mitigate potential zoonotic risks..." (lines 46-48) The conclusion in the main text has also been revised to be more cautious.
13.	All micrographs should include magnification or scale bars directly on the figure. Figure legends should clearly state:<br>• Animal species,<br>• Tissue type,<br>• Diagnostic method used,<br>• Whether the sample was MAP-positive, M. bovis-positive, or co-infected.	We have addressed this across all figures. As requested, we have added scale bars. The figure legends have been expanded to clearly state the animal species, tissue type, diagnostic method, and MAP/*M. bovis* status of the representative sample shown.
14.	While the One Health implications are relevant, the conclusions regarding zoonotic risk and links to human disease should be moderated, as no human or environmental samples were analyzed.	We fully agree. We have moderated the language throughout the manuscript, especially in the Abstract, Discussion (Section 4.3), and Conclusion. The conclusion now focuses on the endemic presence in livestock and the need for surveillance, with the potential public health risk framed as a hypothesis to be tested in future

		research, rather than a confirmed finding (Lines 253-261).
15.	Overall language is clear, but some sentences are dense and could be simplified for readability.	We have carefully reviewed the manuscript and simplified several sentences for clarity and flow, particularly in the Introduction and Discussion sections. We believe this has improved the overall readability.
16.	Ensure consistent formatting of species names (Mycobacterium avium subsp. paratuberculosis, M. bovis).	The entire manuscript has been checked, and species names are now formatted consistently. The full species name is given at first mention, and the standard abbreviation (*M. avium* subsp. *paratuberculosis*, *M. bovis*) is used thereafter.
17.	Use consistent terms for sample types (e.g., "tissue samples" vs "intestinal and MLN samples")	We have standardized the terminology. "Tissue samples" is now used as the general term, and "intestinal tissues" and "mesenteric lymph nodes (MLN)" are used when specifying the precise sample type.
18.	Clarify whether "seroprevalence" refers strictly to iELISA results.	Thank you for pointing this out. We have clarified in the text that "seroprevalence" specifically refers to the results obtained from the iELISA.
19.	Consider adding "One Health" and "abattoir surveillance" as keywords.	These are excellent keywords that reflect the study's scope. We have added "One Health" and "Abattoir surveillance" to the keyword list.
20.	The conclusion currently overstates the implications. It should emphasize:<br>• Endemic presence in livestock,<br>• Need for improved surveillance,<br>• Future integration with human and environmental data	We have rewritten the conclusion as suggested. It now reads:<br>This study confirms the endemicity of MAP in bovines from Peshawar, Pakistan, with a higher infection burden in cattle than buffaloes. The multimodal approach provided a robust epidemiological assessment for the region. The detection of *M. bovis* in the same livestock population underscores the need for enhanced surveillance of zoonotic pathogens. These findings highlight the critical importance of abattoir-based monitoring and the implementation of evidence-based control strategies to improve

		livestock productivity. Future research should integrate human and environmental sampling to fully elucidate the One Health dimensions of mycobacterial infections in this setting (Lines 253-260)

Re: Spectrum02890-25R1 (Molecular Epidemiology and Pathological Burden of Mycobacterium avium subsp. paratuberculosis in Staple livestock from Pakistan: Implications for Zoonotic Surveillance and Livestock Productivity)

Dear Dr. Farhan Anwar Khan:

Thank you for the privilege of reviewing your work. Below you will find my comments, instructions from the Spectrum editorial office, and the reviewer comments.

Please return the manuscript within 60 days; if you cannot complete the modification within this time period, please contact me. If you do not wish to modify the manuscript and prefer to submit it to another journal, notify me immediately so that the manuscript may be formally withdrawn from consideration by Spectrum.

Revision Guidelines
To submit your modified manuscript, log into the submission site at https://spectrum.msubmit.net/cgi-bin/main.plex. Go to Author Tasks and click the appropriate manuscript title to begin. The information you entered when you first submitted the paper will be displayed; update this as necessary. Note the following requirements:

• Upload point-by-point responses to the issues raised by the reviewers in a file named "Response to Reviewers," NOT in your cover letter.
• Upload a compare copy of the manuscript (without figures) as a "Marked-Up Manuscript" file.
• Upload a clean .DOC/.DOCX version of the revised manuscript and remove the previous version.
• Each figure must be uploaded as a separate, editable, high-resolution file (TIFF or EPS preferred), and any multipanel figures must be assembled into one file.
• Any supplemental material intended for posting by ASM should be uploaded with their legends separate from the main manuscript. You can combine all supplemental material into one file (preferred) or split it into a maximum of 10 files with all associated legends included.

For complete guidelines on revision requirements, see our Submission and Review Process webpage. Submission of a paper that does not conform to guidelines may delay acceptance of your manuscript.

Data availability: ASM policy requires that data be available to the public upon online posting of the article, so please verify all links to sequence records, if present, and make sure that each number retrieves the full record of the data. If a new accession number is not linked or a link is broken, provide Spectrum production staff with the correct URL for the record. If the accession numbers for new data are not publicly accessible before the expected online posting of the article, publication may be delayed; please contact production staff (Spectrum@asmusa.org) immediately with the expected release date.

Publication Fees: For information on publication fees and which article types are subject to charges, visit our website. If your manuscript is accepted for publication and any fees apply, you will be contacted separately about payment during the production process; please follow the instructions in that e-mail. Arrangements for payment must be made before your article is published.

ASM Membership: Corresponding authors may join or renew ASM membership to obtain discounts on publication fees. Need to upgrade your membership level? Please contact Customer Service at Service@asmusa.org.

The ASM Journals program strives for constant improvement in our submission and publication process. Please tell us how we can improve your experience by taking this quick Author Survey.

Thank you for submitting your paper to Spectrum.

Sincerely,
Michael Owusu
Editor
Microbiology Spectrum

Reviewer #1 (Comments for the Author):

In the methods part, the authors need to cite the references for sample collection, and should point out all the samples collection are based on biosafety handling and institutional biosafety guidelines.

Reviewer #2 (Comments for the Author):

The authors have addressed my questions and most of my concerns satisfactorily. However, I would have appreciated the inclusion of sequencing of PCR amplicons for confirmation.

The manuscript still requires careful language editing, as there are instances of repetition and minor inconsistencies. For example, in Lines 120-121, "–20{degree sign}C" is repeated and can be corrected.

I recommend that the authors review the manuscript thoroughly, paying close attention to each sentence and making the necessary language revisions.

Responses to Comments

We are grateful to the reviewers and the editor for their insightful and constructive comments, which have significantly strengthened our manuscript. We have carefully addressed each point raised. Below is our point-by-point response. All changes in the manuscript have been highlighted.

S.No	Reviewer 01 Comments	Responses
1.	In the methods part, the authors need to cite the references for sample collection, and should point out all the samples collection are based on biosafety handling and institutional biosafety guidelines.	Thank you for this important suggestion. We have now added appropriate references (23,24) for sample collection, given below.<br>Additionally, we have explicitly stated that all sample collection procedures adhered to institutional biosafety guidelines and standard biosafety protocols (Please refer to lines 116-136).<br><br>• Ottardi M, Lechner I, Wang J, Schmitt S, Schneeberger M, Schmid RM, Stephan R, Meylan M. 2024. Seroprevalence of Mycobacterium avium subsp. paratuberculosis in Swiss dairy herds and risk factors for a positive herd status and within-herd prevalence. Front Vet Sci 11:1409694.<br>• Elze J, Liebler-Tenorio E, Ziller M, Köhler H. 2013. Comparison of prevalence estimation of Mycobacterium avium subsp. paratuberculosis infection by sampling slaughtered cattle with macroscopic lesions vs. systematic sampling. Epidemiol Infect 141:1536-44.
	Reviewer 02 Comments	Responses
1.	The authors have addressed my questions and most of my concerns satisfactorily. However, I would have appreciated the inclusion of sequencing of PCR amplicons for confirmation.	We thank the reviewer for this constructive suggestion. We acknowledge that sequencing would provide an additional layer of confirmation. However, due to resource limitations, we were unable to perform sequencing. Instead, we used species-specific primers validated in peer-reviewed studies (Stabel et al. 2020; Bannantine et al. 2021; Waters et al. 2021) and confirmed primer specificity via NCBI BLAST before PCR (Please

		refer to Table 2).We have now added a sentence in the limitations section (Section 4.4) acknowledging the absence of sequencing as a limitation and recommending it for future studies (Please refer to lines 264-265).
2.	The manuscript still requires careful language editing, as there are instances of repetition and minor inconsistencies. For example, in Lines 120-121, "−20{degree sign}C" is repeated and can be corrected.	We have thoroughly copy-edited the manuscript. The repetition of "−20°C" in lines 121–122 has been corrected. Other minor inconsistencies and redundancies throughout the text have been addressed.
3.	I recommend that the authors review the manuscript thoroughly, paying close attention to each sentence and making the necessary language revisions.	We have carefully re-reviewed the entire manuscript line by line. Grammar, syntax, and clarity have been improved. We believe the manuscript now meets the language standards expected for publication.

Re: Spectrum02890-25R2 (Molecular Epidemiology and Pathological Burden of Mycobacterium avium subsp. paratuberculosis in Staple livestock from Pakistan: Implications for Zoonotic Surveillance and Livestock Productivity)

Dear Dr. Farhan Anwar Khan:

Thank you for the privilege of reviewing your work. Below you will find my comments, instructions from the Spectrum editorial office, and the reviewer comments.

You have addressed the comments from the reviewers assigned to this manuscript. As editor, I would like you to make some few modification before the paper can formally be accepted:
Line 202: Authors should include a detailed table of the risk factor analysis results. They should indicate if any confounders were included in the analysis. Again its not clear whether the risk factor analysis was done based on the PCR, ELIZA or ZN results? This should be clarified

Please return the manuscript within 60 days; if you cannot complete the modification within this time period, please contact me. If you do not wish to modify the manuscript and prefer to submit it to another journal, notify me immediately so that the manuscript may be formally withdrawn from consideration by Spectrum.

Revision Guidelines
To submit your modified manuscript, log into the submission site at https://spectrum.msubmit.net/cgi-bin/main.plex. Go to Author Tasks and click the appropriate manuscript title to begin. The information you entered when you first submitted the paper will be displayed; update this as necessary. Note the following requirements:

• Upload point-by-point responses to the issues raised by the reviewers in a file named "Response to Reviewers," NOT in your cover letter.
• Upload a compare copy of the manuscript (without figures) as a "Marked-Up Manuscript" file.
• Upload a clean .DOC/.DOCX version of the revised manuscript and remove the previous version.
• Each figure must be uploaded as a separate, editable, high-resolution file (TIFF or EPS preferred), and any multipanel figures must be assembled into one file.
• Any supplemental material intended for posting by ASM should be uploaded with their legends separate from the main manuscript. You can combine all supplemental material into one file (preferred) or split it into a maximum of 10 files with all associated legends included.

For complete guidelines on revision requirements, see our Submission and Review Process webpage. Submission of a paper that does not conform to guidelines may delay acceptance of your manuscript.

Data availability: ASM policy requires that data be available to the public upon online posting of the article, so please verify all links to sequence records, if present, and make sure that each number retrieves the full record of the data. If a new accession number is not linked or a link is broken, provide Spectrum production staff with the correct URL for the record. If the accession numbers for new data are not publicly accessible before the expected online posting of the article, publication may be delayed; please contact production staff (Spectrum@asmusa.org) immediately with the expected release date.

Publication Fees: For information on publication fees and which article types are subject to charges, visit our website. If your manuscript is accepted for publication and any fees apply, you will be contacted separately about payment during the production process; please follow the instructions in that e-mail. Arrangements for payment must be made before your article is published.

ASM Membership: Corresponding authors may join or renew ASM membership to obtain discounts on publication fees. Need to upgrade your membership level? Please contact Customer Service at Service@asmusa.org.

The ASM Journals program strives for constant improvement in our submission and publication process. Please tell us how we can improve your experience by taking this quick Author Survey.

Thank you for submitting your paper to Spectrum.

Sincerely,
Michael Owusu
Editor
Microbiology Spectrum

Responses to Comments

We sincerely thank the Editor for the positive evaluation and for providing us with the opportunity to address the remaining concerns regarding our manuscript. We greatly appreciate the thorough review process and the constructive feedback provided by both the reviewers and the editorial team. Below, we provide comprehensive responses to each of the specific comments raised and have incorporated the requested modifications into the revised manuscript.

S.No	Editor Comments	Responses
1.	You have addressed the comments from the reviewers assigned to this manuscript. As editor, I would like you to make some few modifications before the paper can formally be accepted:	We gratefully acknowledge the Editor's positive assessment of our previous revisions and appreciate the opportunity to further strengthen our manuscript through these additional modifications. All suggested changes have been carefully implemented to enhance the clarity, transparency, and scientific rigor of our work.
2.	Line 202: Authors should include a detailed table of the risk factor analysis results.	In response to this valuable suggestion, we have revisited a statistical analysis and have now incorporated Table **7** (highlighted in the revised manuscript) which presents detailed risk factor analysis results, including adjusted odds ratios, 95% confidence intervals, and p-values for all variables assessed. The table has been placed within the Results section (lines 217-226) to facilitate reader comprehension.
3.	They should indicate if any confounders were included in the analysis.	We have explicitly addressed the issue of confounding in our revised manuscript. Age and sex were evaluated as potential confounders using a change-in-estimate criterion of ≥10% but were not retained in the final model as they did not substantially alter the effect estimates and were not independently associated with the outcome. This methodology has been incorporated into the Statistical Analysis section (lines 157-176).
4.	Again its not clear whether the risk factor analysis was done based on the PCR, ELIZA or ZN results? This should be clarified	We appreciate and acknowledge the comment regarding this important clarification. We have now clearly specified that iELISA seropositivity (S/P ratio ≥0.4) served as the primary dependent variable for the multivariable logistic regression analysis. The rationale for this choice includes: (1) serology captures infection status across all disease stages, (2) it provided the largest sample size (n=350) for adequate statistical power, and (3) it aligns with standard epidemiological approaches for MAP risk factor assessment. ZN staining and PCR results were analyzed descriptively and used for diagnostic confirmation (particularly PCR), but were not included as primary outcomes in the risk factor model due to smaller sample sizes (PCR: n=200) and the focal nature of tissue-based detection. This clarification has been added to the Statistical Analysis section (lines 157-166).

Re: Spectrum02890-25R3 (Molecular Epidemiology and Pathological Burden of Mycobacterium avium subsp. paratuberculosis in Staple livestock from Pakistan: Implications for Zoonotic Surveillance and Livestock Productivity)

Dear Dr. Farhan Anwar Khan:

Your manuscript has been accepted, and I am forwarding it to the ASM production staff for publication. Your paper will first be checked to make sure all elements meet the technical requirements. ASM staff will contact you if anything needs to be revised before copyediting and production can begin. Otherwise, you will be notified when your proofs are ready to be viewed.

Data Availability: ASM policy requires that data be available to the public upon online posting of the article, so please verify all links to sequence records, if present, and make sure that each number retrieves the full record of the data. If a new accession number is not linked or a link is broken, provide production staff with the correct URL for the record. If the accession numbers for new data are not publicly accessible before the expected online posting of the article, publication may be delayed; please contact ASM production staff immediately with the expected release date.

Publication Fees: For information on publication fees and which article types have charges, please visit our website. We have partnered with Copyright Clearance Center (CCC) to collect author charges. If fees apply to your paper, you will receive a message from no-reply@copyright.com with further instructions. For questions related to paying charges through RightsLink, please contact CCC at ASM_Support@copyright.com or toll free at +1-877-622-5543. CCC makes every attempt to respond to all emails within 24 hours.

ASM Membership: Corresponding authors may join or renew ASM membership to obtain discounts on publication fees. Need to upgrade your membership level? Please contact Customer Service at Service@asmusa.org.

Embargo Policy: A press release may be issued as soon as the manuscript is posted on the Spectrum Latest Articles webpage. The corresponding author will receive an email with the subject line "ASM Journals Author Services Notification" when the article is available online.

Cover Image Submissions: If you would like to submit a potential Cover Image, please email a file and a short legend to spectrum@asmusa.org. Please note that we can only consider images that (i) the authors created or own and (ii) have not been previously published. By submitting, you agree that the image can be used under the same terms as the published article. Image File requirements: TIF/EPS, 7.5 inches wide by 8.25 inches tall (at least 2,250 pixels wide by 2,475 pixels tall), minimum 300 dpi resolution (600 dpi preferred), RGB, and no figure elements, e.g., arrows or panel labels. The legend should be a short description of the image, 1-2 sentences recommended. Please use the ASM Journals Cover Image Sizing Tool (https://journals.asm.org/pb-assets/html/asm-cover-sizing-tool.html) to ensure that your proposed cover image meets our size requirements.

The ASM Journals program strives for constant improvement in our submission and publication process. Please tell us how we can improve your experience by taking this quick Author Survey.

Thank you for submitting your paper to Spectrum.

Sincerely,
Michael Owusu
Editor
Microbiology Spectrum
